# An Atypical Presentation of Chronic Atrophic Gastritis: Hemolytic Anemia and Mesenteric Panniculitis

**DOI:** 10.1155/2017/5498034

**Published:** 2017-07-03

**Authors:** Zurab Azmaiparashvili, Vinicius M. Jorge, Catiele Antunes

**Affiliations:** Albert Einstein Medical Center, Philadelphia, PA, USA

## Abstract

Microangiopathic hemolytic anemia (MAHA) requires an aggressive approach since primary thrombotic microangiopathy syndromes such as thrombotic thrombocytopenic purpura (TTP) can progress rapidly to a fatal outcome. Differential diagnosis can be challenging even for an experienced hematologist. We present a case of a 52-year-old male who presented with symptoms of mesenteric panniculitis and showed signs of MAHA. His condition was attributed to severe vitamin B12 deficiency secondary to chronic atrophic gastritis and initiation of appropriate therapy was met with complete resolution of symptoms and normalization of hematologic parameters.

## 1. Introduction

Thrombotic microangiopathies are a diverse group of disorders that may be congenital or acquired, present in childhood or in adults, and manifest an acute or a more gradual progressive course [1]. Clinically they are characterized by thrombocytopenia, microangiopathic hemolytic anemia (MAHA), and extremely elevated serum lactate dehydrogenase (LDH) levels with or without signs of end-organ damage [1, 2]. Differential diagnosis can be challenging as primary thrombotic microangiopathy syndromes such as thrombotic thrombocytopenic purpura (TTP) can progress rapidly to a fatal outcome without prompt initiation of effective therapy [3]. In this article, we discuss a case of severe vitamin B12 deficiency presenting as concurrent microangiopathic hemolytic anemia and mesenteric panniculitis.

## 2. Case Report

A 52-year-old Hispanic male with past medical history significant for essential hypertension, gastroesophageal reflux disease (GERD), and recently diagnosed anemia attributed to iron deficiency presented to the Emergency Department with three weeks' history of left flank pain. Pain was described as severe, sharp, and constant in nature, made worse with prolonged fasting or following a meal. No recognizable alleviating factors were reported. Associated symptoms included nausea, vomiting of nonbloody, nonbilious secretions, and retrosternal burning. There was no hematemesis, melena, perrectal bleeding, or change in bowel habits. Patient also reported progressive weakness and fatigue for two weeks and weight loss of approximately ten pounds over the last two months. Patient was prescribed omeprazole and iron supplements for newly diagnosed anemia and abdominal pain by his primary care physician. He denied taking other prescription or over-the-counter medications, herbal supplements, or illicit drugs. Patient denied alcohol or tobacco use. There was no personal or family history of solid or hematologic malignancies or bleeding or thrombotic events.

Patient was afebrile on admission with normal hemodynamic parameters. Physical examination revealed conjunctival pallor and scleral icterus. Cardiovascular and pulmonary examination were within normal limits. Abdominal examination revealed mild to moderate left flank and epigastric tenderness with no rebound or involuntary guarding. There was no palpable hepatosplenomegaly or peripheral lymphadenopathy. The rest of the examination was largely unremarkable.

Complete blood count (CBC) on admission revealed hemoglobin 6.3 g/dL, hematocrit 17.9%, white blood cell (WBC) count 4,100/*μ*L, platelet count 88,000/*μ*L, and reticulocyte count 0.86%. Mean corpuscular volume (MCV) was 96.2 fL and red blood cell distribution width (RDW) was 23.4%. Basic metabolic panel (BMP) showed normal serum electrolyte concentrations and normal renal function with creatinine 0.8 mg/dL and blood urea nitrogen (BUN) 14 mg/dL. Liver function tests (LFTs) on admission revealed elevated total bilirubin concentration of 4.5 mg/dL with direct fraction of 0.5 mg/dL and mildly elevated alanine aminotransferase (ALT) and aspartate aminotransferase (AST) levels of 67 IU/L and 165 IU/L, respectively. Coagulation profile showed international normalized ratio (INR) of 1.2 and partial thromboplastin time (PTT) of 25.4 seconds. Direct antiglobulin test and antibody screen were negative. Lactate dehydrogenase (LDH) was 6,235 IU/L while haptoglobin was <8.0 mg/dL.

**Figure 1:**
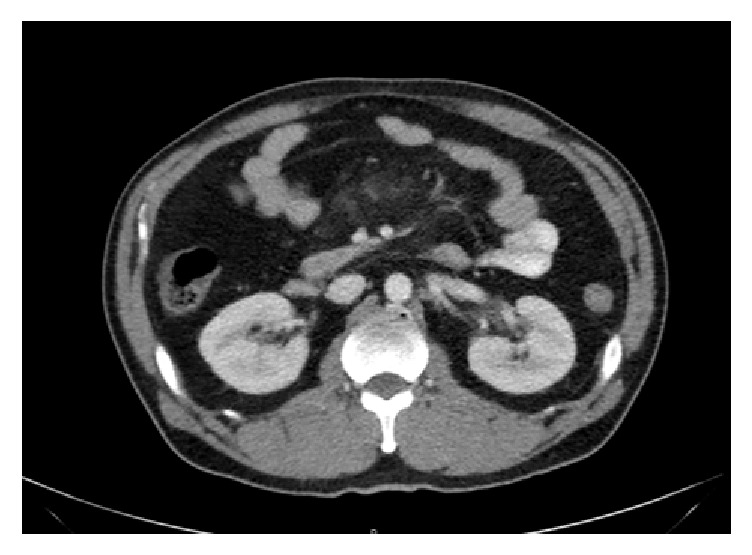
CT scan of the abdomen and pelvis showing inflammatory fat stranding in the small bowel mesentery (axial view).

**Figure 2:**
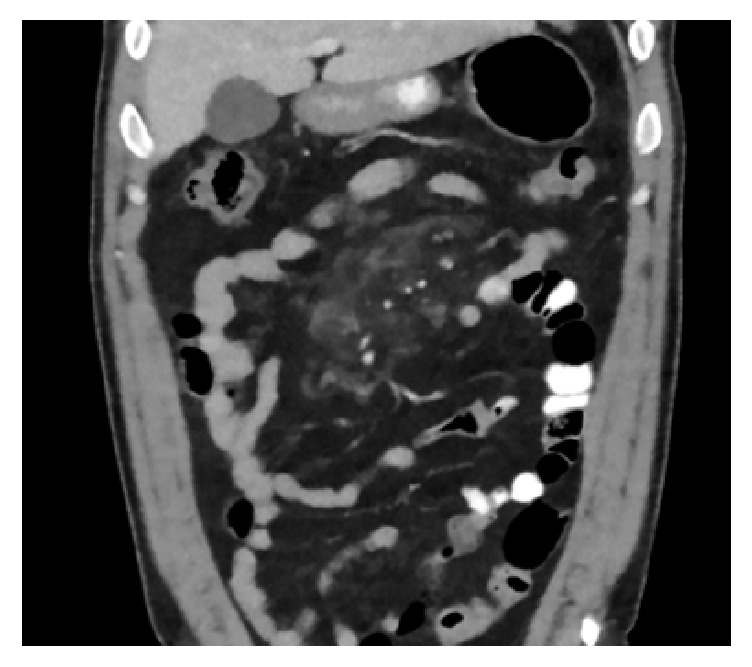
Redemonstration of inflammatory fat stranding in the small bowel mesentery (coronal view).

Computed tomography (CT) scan of the abdomen and pelvis showed acute mesenteric inflammation involving central small bowel mesentery, compatible with the diagnosis of mesenteric panniculitis (see Figures 1 and 2).

**Figure 3:**
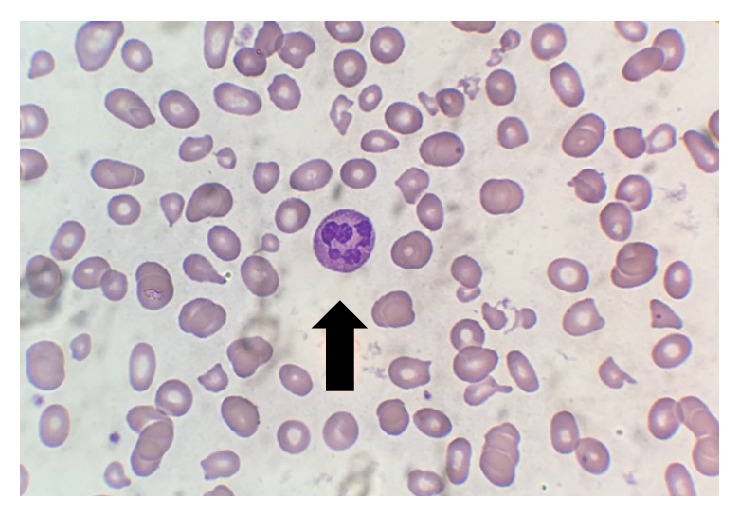
Peripheral blood smear showing poikilocytosis, schistocytosis, thrombocytopenia, and a hypersegmented neutrophil (arrow).

**Figure 4:**
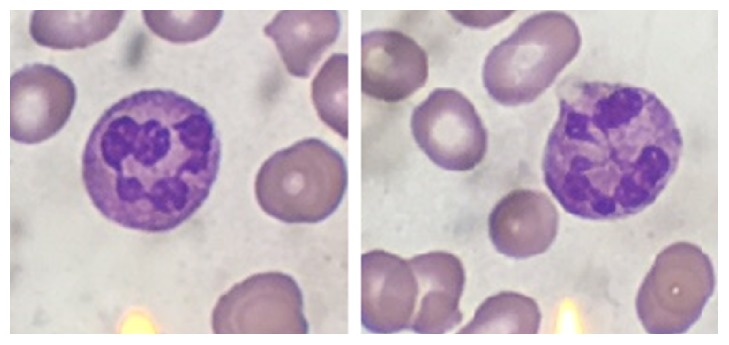
Magnified view of hypersegmented neutrophils.

Peripheral blood smear revealed an abundance of schistocytes and occasional hypersegmented neutrophils (see Figures 3 and 4). This, along with the initial laboratory findings, was concerning for an atypical presentation of thrombotic thrombocytopenic purpura (TTP).

**Figure 5:**
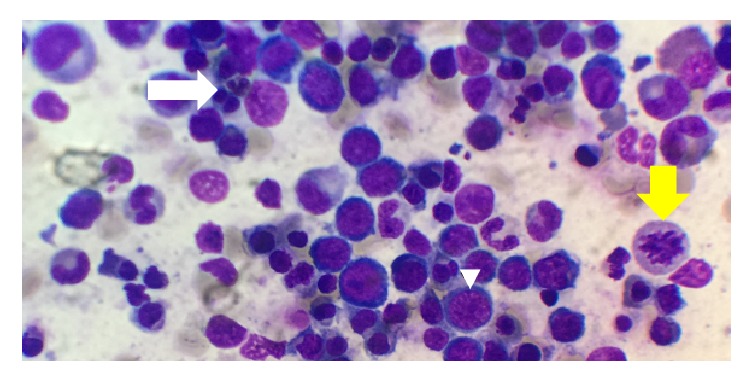
Bone marrow smear showing multinucleated red cell (white arrow), typical megaloblast (arrowhead), and mitotic phase cell (yellow arrow).

**Figure 6:**
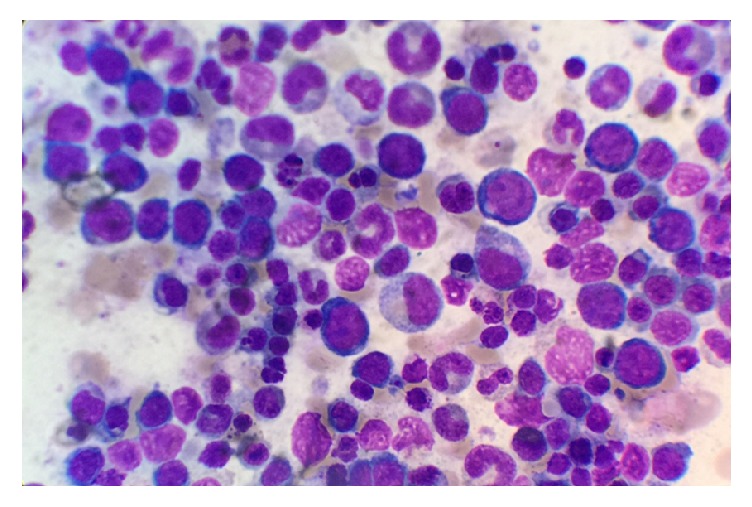
Bone marrow smear showing erythroid hyperplasia with dysplastic features and megaloblastic changes.

At this point, the plan was to start urgent plasmapheresis. However, within the next hour, serum vitamin B12 level was reported at <109 pg/dL (normal range: 213–816 pg/dL) with serum homocysteine level of 86.8 *μ*mol/L (normal range: 5.1–15.4 *μ*mol/L). Plasmapheresis plans were stopped. Bone marrow smear and biopsy were performed confirming erythroid hyperplasia and megaloblastic anemia (see Figures 5 and 6). Cyanocobalamin repletion was promptly initiated.

Over the next 2 days, hemoglobin remained stable, reticulocyte count increased to 1.17%, and major improvement in the degree of hemolysis was noted. Furthermore, abdominal pain resolved and patient was tolerating regular diet. Gastroenterology team recommended upper endoscopy and considered mesenteric panniculitis reactive to underlying hematologic disorder. Late during the course of this admission, the sent-out laboratory tests showed methylmalonic acid level of 4080 nmol/L (normal range: 87–318 nmol/L), while the serologic tests for Intrinsic Factor Blocking Antibody and Anti-Gastric Parietal Cell Antibody were strongly positive. Together, these findings suggested autoimmune chronic atrophic gastritis as the underlying etiology of current presentation.

**Figure 7:**
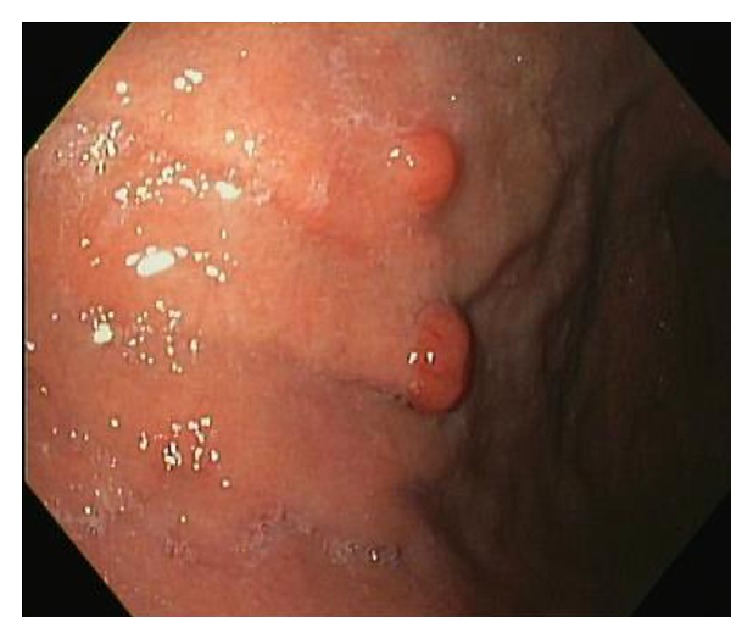
EGD showing two firm sessile polyps.

Esophagogastroduodenoscopy (EGD) was performed as outpatient showing two large, sessile polyps in the gastric body (see Figure 7) that were completely removed. The remainder of the EGD was within normal limits. Gastric polyp histologic examination was positive for well-differentiated neuroendocrine tumor and background changes of chronic atrophic gastritis.

Patient continues to receive cyanocobalamin replacement therapy as outpatient. Vitamin B12 replacement has been met with complete normalization of hematologic parameters and resolution of symptoms of mesenteric panniculitis.

This is the first case report of vitamin B12 deficiency secondary to chronic autoimmune gastritis, presenting with concurrent microangiopathic hemolytic anemia and mesenteric panniculitis.

## 3. Discussion

TTP has been classically diagnosed based on a pentad of MAHA, thrombocytopenia, neurologic abnormalities, renal dysfunction, and fever [3, 4]. However, with the introduction of plasmapheresis as the effective treatment modality, presumptive diagnosis is commonly made on the basis of MAHA and thrombocytopenia without an apparent cause [3, 5]. As a result, up to 10% of patients diagnosed with TTP and receiving plasmapheresis may be later found to have an alternate diagnosis [6], underlining the importance of continuous reevaluation.

The most common symptoms of TTP at presentation are generalized weakness, abdominal pain, nausea, and vomiting [3]. With suggestive clinical symptoms and characteristic laboratory findings, it is not surprising that our patient was presumptively diagnosed with TTP and planned for initiation of plasmapheresis. However, the possibility of vitamin B12 deficiency was also entertained in view of relatively mild thrombocytopenia, extremely elevated LDH level, and presence of hypersegmented neutrophils on blood smear.

Vitamin B12 deficiency causes dyssynchrony between nuclear and cytoplasmic maturation, resulting in ineffective erythropoiesis, intramedullary hemolysis, and characteristic features of megaloblastic anemia on blood smear: macrocytosis and hypersegmented neutrophils [7]. Other less common hematologic findings associated with vitamin B12 deficiency include thrombocytopenia, pancytopenia, and hemolytic anemia [8]. Furthermore, vitamin B12 deficiency has been implicated as a cause of thrombotic microangiopathy mimicking TTP [9–13]. The mechanism of thrombotic microangiopathy and resultant MAHA is not fully understood, but the presence of hyperhomocysteinemia has been suggested as the plausible link between vitamin B12 deficiency and endothelial dysfunction and microvascular thrombi formation [14, 15].

Mesenteric panniculitis is a relatively uncommon benign condition characterized by varying degrees of inflammation, fat necrosis, and fibrosis of the small bowel mesentery [16]. It may occur spontaneously or in association with other disorders, including abdominal trauma, hematologic and solid organ malignancies, and autoimmune conditions [16, 17]. The majority of cases are diagnosed incidentally during the work-up of an unrelated condition [17]. The most common symptoms in clinically apparent cases are abdominal pain, nausea and vomiting, change in bowel habits, and weight loss [18]. Confirmation of diagnosis generally requires histologic proof of mesenteric panniculitis; however, diagnosis is often suggested by typical radiologic findings [16, 17]. Treatment options are varied, nonstandardized, and typically reserved for symptomatic patients [16, 18]. A myriad of anti-inflammatory and hormonal therapies, including systemic corticosteroids and tamoxifen, alone or in combination, have been used with variable success. Most cases tend to resolve spontaneously with no intervention [16–18].

Mesenteric panniculitis has been reported in association with vitamin B12 deficiency and chronic atrophic gastritis [19]. The patient was treated with prolonged course of systemic corticosteroids with complete symptomatic and radiologic remission. We did not consider treatment of mesenteric panniculitis in our patient as the disease process was assumed to be secondary to concurrent hematologic abnormalities. To our knowledge, there have been no case reports of concurrent mesenteric panniculitis and thrombotic microangiopathy secondary to vitamin B12 deficiency.

Gastric carcinoid tumors, also called gastroenteropancreatic neuroendocrine tumors, comprise approximately 10% to 30% of all carcinoid tumors [20]. There are four different types of gastric carcinoid tumors with type 1 comprising the majority of cases [20, 21]. Type 1 carcinoid tumors are found in association with chronic atrophic gastritis and achlorhydria and are usually multiple, nodular, or polypoid in nature and less than 1 cm in size [20]. Endoscopic resection is the treatment of choice for type 1 gastric carcinoid tumors and they generally follow a benign course with >95% 5-year survival rate [20, 21].

## 4. Conclusion

Thrombotic microangiopathies are a diverse group of disorders that share microangiopathic hemolytic anemia and thrombocytopenia in common. Vitamin B12 deficiency may present in a similar fashion and pose a diagnostic and therapeutic challenge to the clinicians. We propose exploring further work-up for exclusion of vitamin B12 deficiency as the cause of thrombotic microangiopathy, especially for cases with extremely elevated LDH levels (>2500 IU/L), low reticulocyte counts, mild to moderate thrombocytopenia (>50,000/*μ*L), and/or atypical findings on blood smear such as hypersegmented neutrophils.
